# Validation of the Patient Health Questionnaire‐2 (PHQ‐2) in Detecting Depression Among Older Adults

**DOI:** 10.1002/agm2.70089

**Published:** 2026-06-15

**Authors:** Francesco Salis, Maristella Belfiori, Alessandro Ferrau, Eleonora Bernardini, Emanuele Concas, Anna Maria Lugas, Antonella Mandas

**Affiliations:** ^1^ Department of Medical Sciences, and Public Health University of Cagliari Cagliari Italy; ^2^ Department of Biomedical Sciences University of Cagliari Cagliari Italy; ^3^ University Hospital “Azienda Ospedaliero‐Universitaria” of Cagliari Cagliari Italy

**Keywords:** depression, geriatrics, screening

## Abstract

**Objectives:**

Depression screening tools like the Patient Health Questionnaire‐2 (PHQ‐2) are commonly used to detect depression. However, its performance in elderly populations has been less extensively studied compared to its broader adult use. This study evaluates the PHQ‐2's ability to screen for depression in older adults.

**Methods:**

A cross‐sectional study was conducted by enrolling 320 elderly individuals (≥ 65 years) from a geriatric outpatient service from May 2023 to July 2024. The sample was randomly split into a validation group and a test group. The discriminatory power of PHQ‐2 was evaluated by comparing it to the Geriatric Depression Scale (GDS).

**Results:**

The PHQ‐2 demonstrated good discriminatory ability, with a Receiver Operating Characteristic Area Under the Curve (AUC) of 0.802 (95% CI: 0.703–0.901) in the validation group. The optimal cutoff for the PHQ‐2 was 3 points, yielding a sensitivity of 71% and accuracy of 78%. The model showed high specificity (80%) and negative predictive value (93%), suggesting that few depressed individuals would go undetected. These findings were consistent in the test group (AUC: 0.789).

**Conclusions:**

The PHQ‐2 is a reliable and efficient tool for screening depression in older adults, with strong diagnostic performance and ease of use. This study supports its application in primary care settings, facilitating timely referrals for further evaluation.

## Graphical abstract

PHQ‐2 effectively screens for geriatric depression with the optimal cut‐off of 3 points.

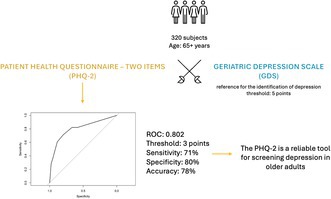

## 1 Introduction

Depression in the older population is a prevalent and debilitating mental health condition, often associated with significant mortality and morbidity, such as declined physical and cognitive functions, increased dependency, and reduced quality of life [1]. It is estimated that depressive symptoms affect approximately 10%–15% of older adults, while major depressive disorder is less common but clinically significant [1, 2, 3, 4]. Effective screening tools are essential for early identification and intervention. Among the tools commonly used in primary care and geriatric settings, the Patient Health Questionnaire‐2 (PHQ‐2) has gained attention due to its brevity and ease of administration. Comprising just two questions that assess the frequency of depressed mood and anhedonia over the past 2 weeks, the PHQ‐2 serves as an initial screening step before more detailed evaluation with the PHQ‐9. Other screening tools have been developed specifically for the geriatric population, such as the Geriatric Depression Scale (GDS) [5]. It represents a cornerstone in geriatric depression assessment, although it requires the absence of severe cognitive impairment and more time to administer. Nonetheless, it has been demonstrated that rating scales, including the GDS, can discriminate depressed and non‐depressed older individuals [3]. Also, depression prevalence rates based on rating scales are similar to those diagnosed with specific clinical criteria. In summary, GDS can be considered a valid tool to assess depression in older adults [6, 7]. A recent study comparing depression prevalence assessed with GDS and structured clinical interviews concluded that the 5‐point threshold may overestimate prevalence [2]. As for PHQ‐2, its discriminative power in detecting depression among elderly individuals has been the subject of ongoing research. Studies suggest that its sensitivity and specificity are robust across various populations, including those with chronic illnesses [8]. A systematic review and meta‐analysis published in 2020 affirmed that its discriminatory capacity for major depression is similar to the full PHQ‐9 [4], as previously suggested by other studies [9], all conducted in adult populations.

Following this background, this study aims to evaluate the discriminative capacity of the PHQ‐2 in identifying depression among older adults.

## 2 Methods

This investigation took the form of a cross‐sectional study, encompassing individuals consecutively evaluated at the Geriatric Service of the University Hospital of Monserrato, Cagliari, Italy, from May 2023 to July 2024. Exclusion criteria included: (i) individuals under the 65 years of age, (ii) those with severe cognitive impairment, defined as a mini‐mental state examination (MMSE) score below 15, and (iii) individuals who did not provide informed consent. No data are available on the number of potentially eligible individuals excluded for the abovementioned reasons.

In consideration of a 95% confidence level, a 5% confidence interval, an anticipated prevalence (P) of 0.2 [2, 3], a *Z*‐score (z) of 1.96, and an error margin (e) of 5%, the requisite sample size (*n*) was calculated to be at least 246 subjects, as calculated by the formula:

$$n=\frac{z^{2}*P\;\left(1-P\right)}{e^{2}}$$

The enrolled subjects were evaluated using the following tools:

○ *GDS* [5]: A 15‐item yes/no tool used to differentiate between depressed and non‐depressed older adults. Scores range from 0–15, with higher scores indicating a greater likelihood of depression. Various thresholds have been proposed; however, the 5‐point cutoff appears to overestimate depression [2], while higher cutoffs, such as 10 points, are generally preferred [6, 7].
○ *PHQ‐2* [8, 9]: A 2‐item depression screening tool that explores the frequency of depressive symptoms over the past 2 weeks. Scores range from 0–6, with higher scores indicating a greater likelihood of depression. The 3‐point threshold has been identified as optimal [8].
○ *MMSE* [10, 11]: A first‐level neurocognitive screening tool. Scores range from 0–30, and lower scores indicate more severe cognitive impairment [12].
○ *Barthel Index* [13]: An index that measures the need for assistance in performing basic activities of daily living, and higher scores indicate greater independence.
○ *Instrumental Activities of Daily Living (IADL)* [14]: An index that measures the need for assistance in performing instrumental activities of daily living, and higher scores indicate greater independence.
○ *Cumulative Illness Rating Scale (CIRS)* [15]: An index assessing the burden and severity of comorbidities across 14 categories.
○ *Multidimensional Prognostic Index (MPI)* [16]: A multidimensional index used to stratify patients by risk of adverse event (low, moderate, and high risk). Its validity has been confirmed across various clinical settings [17, 18, 19, 20, 21], and it has also been validated as a tool for identifying frailty [22].

## 3 Statistical Analysis

The Shapiro–Wilk test was used to assess normal distribution of the variables. The variables were expressed as medians and interquartile ranges (IQRs) or in absolute numbers and percentages (%), where appropriate. Categorial variables were compared with the chi‐squared (*χ* ^2^) test. Continuous variables were compared with the Wilcoxon rank sum test.

We aimed to validate a threshold for the PHQ‐2 that effectively discriminates depression, employing a model of internal validation. The sample was randomly divided into two groups (“validation” and “test”) using statistical software. In detail, randomization was performed using a computer‐generated random sequence implemented in RStudio software. Then, we developed a regression model utilizing the 10‐point cut‐off on the GDS to distinguish between people with and without depression [6, 7]. The PHQ‐2 scores were treated as independent variables in the “validation” sample. Its discriminating performance was measured by the Area Under Receiver Operating Characteristic (ROC) Curve (AUC). Youden's J statistic was applied to identify optimal cut‐off values. The model was subsequently validated using the “test” sample by comparing ROC AUCs.

The results are reported indicating *p*‐values in reference to 95% confidence intervals (95% CI).

RStudio software (2024.09.0 + 375 version) was used for the statistical analysis.

## 4 Results

The study comprised, according to inclusion/exclusion criteria, 320 elderly subjects, of whom 206 (67.5%) were women. The median age was 81 years (IQR: 76–85), with a higher comorbidity burden and an overall moderate risk of adverse event. Anti‐hypertensives were the most frequently prescribed medications. The other characteristics of the enrolled subjects are detailed in Table 1.

**TABLE 1:** Characteristics of the sample.

Variable	Median	IQR
Age (years)	81	76–85
Education (years)	7	5–10
Medications taken (*n*)	6	4–9
GDS	5	3–9
PHQ‐2	0	0–3
MMSE	24	20–27
Barthel Index	86	72–94
IADL	4	2–6
CIRS	30	27–33

GDS scores	*n*	%
GDS < 5	133	41.5
GDS 5–9	124	38.8
GDS ≥ 10	63	19.7

MPI scores	*n*	%
MPI < 0.33 (low risk)	114	35.6
MPI 0.33–0.66 (moderate risk)	182	56.9
MPI ≥ 0.67 (high risk)	24	7.5

Medications taken	*n*	%
Anti‐hypertensive	229	71.6
Statin	146	45.6
Proton Pump Inhibitor	140	43.8
Benzodiazepine	99	30.9
SSRI	32	10.0
SNRI	18	5.6
Other antidepressant	31	9.7
Antipsychotic	26	8.1

Abbreviations: CIRS, Comorbidity Index Rating Scale; GDS, Geriatric Depression Scale; IADL, Instrumental Activities of Daily Living; IQR, Interquartile Range; MMSE, Mini‐Mental State Examination; MPI, Multidimensional Prognostic Intex; PHQ‐2, Patient Health Questionnaire, two items; SNRI, Serotonin and Norepinephrine Reuptake Inhibitors; SSRI, Selective Serotonin Reuptake Inhibitors.

Based on GDS scores, 63 participants (19.7%) had scores ≥ 10, while 257 scored below 10, including 124 individuals who scored between 5–9.

We randomly divided the sample in two subsamples, each containing 160 participants (“validation” and “test” groups). No significant differences were observed in age, sex distribution, GDS scores, and MPI (see Table 2).

**TABLE 2:** Differences between “validation” and “test” group.

Variable	Validation median (IQR)	Test median (IQR)	*p*
Age (years)	80 (8)	81 (9)	0.106
GDS	6 (6)	5 (7)	0.139

Variable	*n*	*n*	*p*
Sex (female)	110	106	0.720
Sex (male)	50	54	
MPI < 0.33	56	58	0.389
MPI 0.33–0.66	95	87	
MPI ≥ 0.67	9	15	

Abbreviations: GDS, Geriatric Depression Scale; IQR, Interquartile Range; MPI, Multidimensional Prognostic Intex.

**FIGURE 1:**
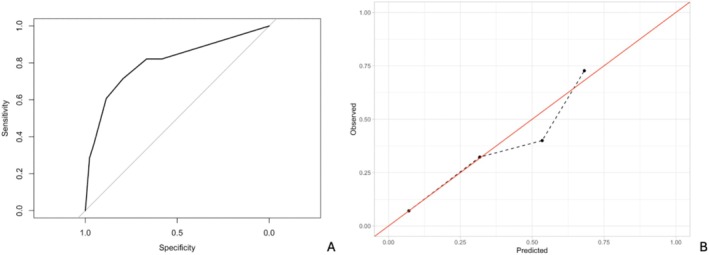
Curves from validation model. (A) ROC AUC; (B) calibration curve.

**FIGURE 2:**
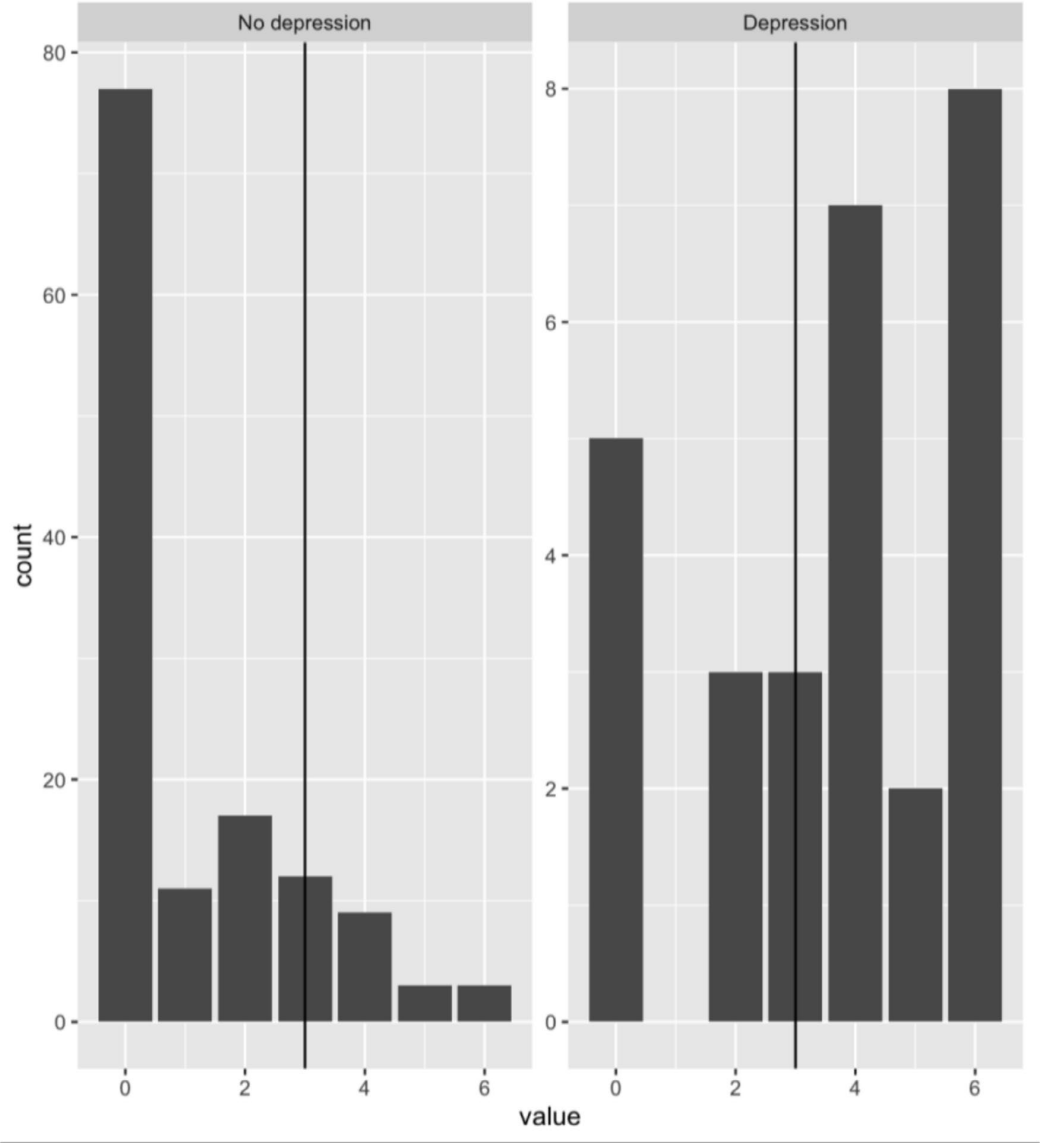
Identification of optimal cut‐off threshold.

In validation group, as outlined in Methods section, referring to the literature on the topic, individuals with GDS scores ≥ 10 were categorized as “depressed,” while those with GDS scores < 10 as “non‐depressed” (model GDS_10). The discriminatory capacity of the PHQ‐2 yielded a ROC AUC of 0.802 (95% CI: 0.703–0.901), as displayed in Figure 1A. The model's calibration is shown in Figure 1B. Youden's J index identify the 3‐point threshold as optimal, with sensitivity: 0.714, specificity: 0.796, and accuracy: 0.781, as displayed in Figure 2. Furthermore, the positive predictive value was 0.426, negative predictive value was 0.929, with a positive likelihood ratio of 3.494, and negative likelihood ratio of 0.360.

**FIGURE 3:**
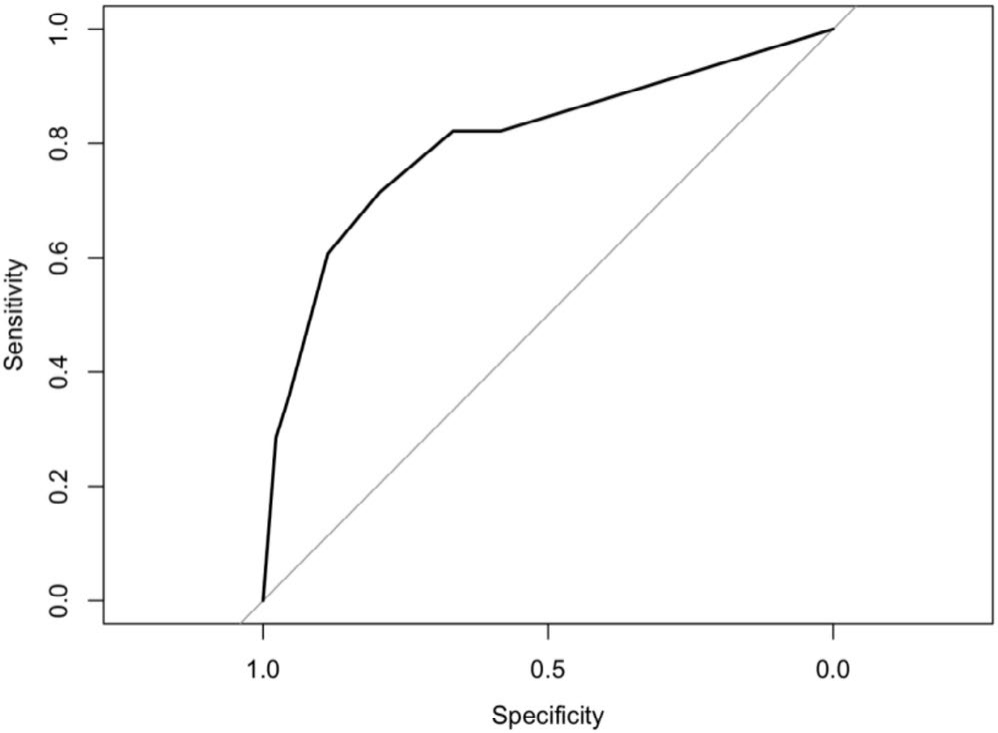
ROC AUC of test model.

Subsequently, we applied the model derived from the validation group to the test group. The analysis revealed a ROC AUC: 0.789 (95% CI: 0.698–0.880), as displayed in Figure 3.

For the sake of completeness, we performed the same analysis on the validation group using a GDS score threshold of 5 (model GDS_5). The analysis produced a ROC AUC of 0.780 (95% CI: 0.714–0.848), although with poorer calibration (see Figure S1A,B). The comparison of the ROC curves for the two models is shown in Figure S2.

## 5 Discussion

This study was aimed at testing the capacity of PHQ‐2 to distinguish between depressed and non‐depressed individuals aged 65 years and older, without severe cognitive deficit, since cognitive impairment could compromise the accuracy of responses to depression questionnaires. The prevalence of depression, based on GDS scores ≥ 10, was approximately 20%, consistently with existing literature on similar populations [1, 2, 3]. Our model demonstrated that the PHQ‐2 possesses good discriminatory ability (AUC: 0.802), and was well‐calibrated, with predicted risks closely aligning with observed risks [23]. When comparing this validation model, obtained with the aforementioned GDS threshold, to a model using a lower GDS threshold, we observed that, though showing a fair AUC (0.780), the latter suffered from poor calibration, underestimating risk among individuals with higher predicted probabilities. Considering Youden's J index, the 3‐point cut‐off provided an optimal sensitivity/specificity balance. The relatively low positive predictive value likely reflects the relatively low prevalence of the condition in our population, as evidenced by the higher positive likelihood ratio. Clinically, it implies that positive findings may include false positives, even though the positive likelihood ratio supports the PHQ‐2's good discriminatory ability. Furthermore, the high specificity and the optimal negative predictive value suggest that few depressed individuals would go undetected by the PHQ‐2. Notably, the identified threshold aligns with findings from the PHQ‐2 validation trial [8], conducted in adults aged 18 years and older, which has previously demonstrated its reliability in other populations [4, 9]. A recent meta‐analysis [9] indicated that the PHQ‐2's discriminatory capacity is similar to that of the PHQ‐9, albeit with the 2‐point threshold. This discrepancy with our results may reflect the significantly lower mean age (49 years) in their sample, without excluding the possibility that our results may suffer from a significantly less numerous sample. It leaves the possibility for future research to review these findings with larger samples. Anyhow, the reliability of our model has been confirmed in the test sample, with an AUC of 0.789.

In conclusion, our study demonstrates that PHQ is a trustworthy and reliable depression screening tool even in its 2‐item form. Its simplicity and rapid administration, requiring less than a minute, make it suitable for use in primary care settings, facilitating timely referrals to geriatricians or psychiatrists for further evaluation. To the best of our knowledge, this study represents one of the first attempts to validate the PHQ‐2 alone in a population of older adults of such advanced age. Clinically, these findings support the use of PHQ‐2 as a practical, time‐efficient tool for early detection of depression in older patients. Compared with previous research, our findings align with studies suggesting the PHQ‐2's discriminative ability is robust, even among older individuals with a higher comorbidity burden [4, 8, 9].

We also acknowledge some limitations. First, depression was identified according to GDS scores. Although it is supported by the literature, more precise results could have been obtained through structured interviews and applying clinical guidelines. Consequently, the use of this scale may have led to misclassification, potentially inflating or underestimating the PHQ‐2's diagnostic performance. In detail, some individuals classified as depressed by the GDS may not meet formal diagnostic criteria for major depressive disorder, while others with clinically relevant depression may not reach the selected threshold. Therefore, results should be interpreted with caution. Second, it should be also noted that some confounding factors could have potentially influenced the PHQ‐2 performance. However, these same factors may also have affected responses on the GDS, which was used as the reference standard. This potential influence should be considered when interpreting the findings, and future research could explore whether diagnostic accuracy varies across subgroups. Moreover, the study only encompassed patients who achieved at least 15 points at MMSE, excluding the most cognitively impaired part of the population, who should be the focus of future research to identify optimal depression screening tools. Lastly, as a monocentric study, the generalizability of our findings to the broader geriatric population may be limited. In detail, individuals attending a specialized geriatric outpatient service may differ from community‐dwelling older adults in terms of health status, healthcare access, and even clinical complexity. Therefore, caution is warranted when extrapolating these results to other settings.

## Author Contributions

**Francesco Salis:** conceptualization, data curation, formal analysis, methodology, writing – original draft. **Maristella Belfiori:** data curation, methodology, writing – review and editing. **Alessandro Ferrau:** investigation, data curation, visualization. **Eleonora Bernardini:** investigation, data curation, visualization. **Emanuele Concas:** investigation, data curation, visualization. **Anna Maria Lugas:** investigation, data curation, visualization. **Antonella Mandas:** conceptualization, methodology, supervision, writing – review and editing.

## Funding

This research did not receive specific funding.

## Ethics Statement

The study was conducted according to the guidelines of the Declaration of Helsinki and approved by an Institutional Review Board (Institutional Review Board of the University of Cagliari, protocol code NP/2022/1382, 30 March 2022).

## Conflicts of Interest

The authors declare no conflicts of interest.

## Supporting information


**Figure S1:** Curves from GDS_5 validation model.
**Figure S2:** Comparison of GDS_10 and GDS_5 ROC curves.

## Data Availability

The data and materials used and/or analyzed during the current study are not publicly available. The are available from the corresponding author (FS) upon reasonable request.
