# Supplementary material for: Validation of the Patient Health Questionnaire‐2 (PHQ‐2) in Detecting Depression Among Older Adults
Source: Aging Med (Milton). 2026 Jun 15;9(3):244–9. doi: 10.1002/agm2.70089 (PMC13347153; doi:10.1002/agm2.70089)

**Patient Health Questionnaire-2 (PHQ-2) in detecting depression among older adults**

**Supplementary Materials**

Francesco Salis^1,2,*^, Maristella Belfiori^1,#^, Alessandro Ferrau^1^, Eleonora Bernardini^1^, Emanuele Concas^1^, Anna Maria Lugas^1^, Antonella Mandas^1,3^

^1^ Department of Medical Sciences, and Public Health – University of Cagliari – Cagliari (Italy)

^2^ Department of Biomedical Sciences – University of Cagliari – Cagliari (Italy)

^3^ University Hospital “Azienda Ospedaliero-Universitaria” of Cagliari – Cagliari (Italy)

* Correspondence: Francesco Salis

Mailing Address: SS 554 bivio Sestu, 09042 Monserrato (Cagliari), Italy

Tel +39 070 6754307

Email f.salis19@studenti.unica.it

# shared first authorship

Supplementary Figure 1 – Curves from GDS_5 validation model


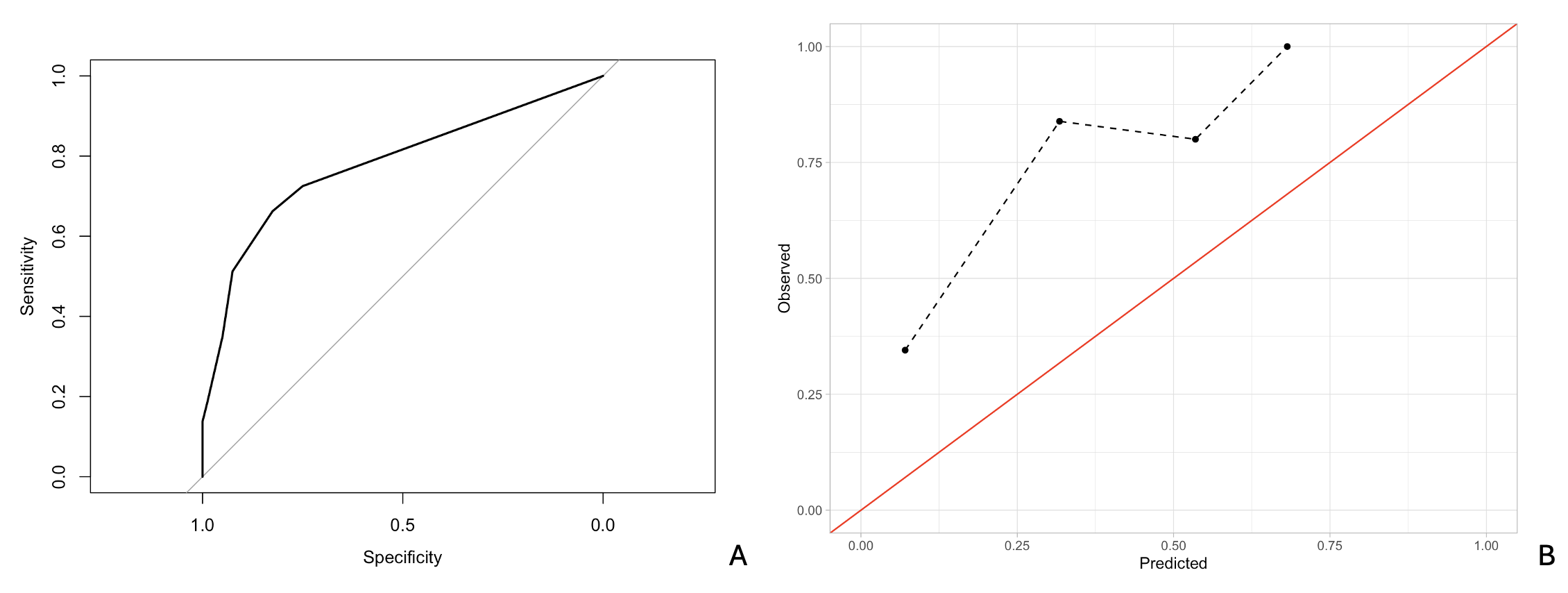


A, ROC AUC; B, calibration curve

Supplementary Figure 2 – Comparison of GDS_10 and GDS_5 ROC curves


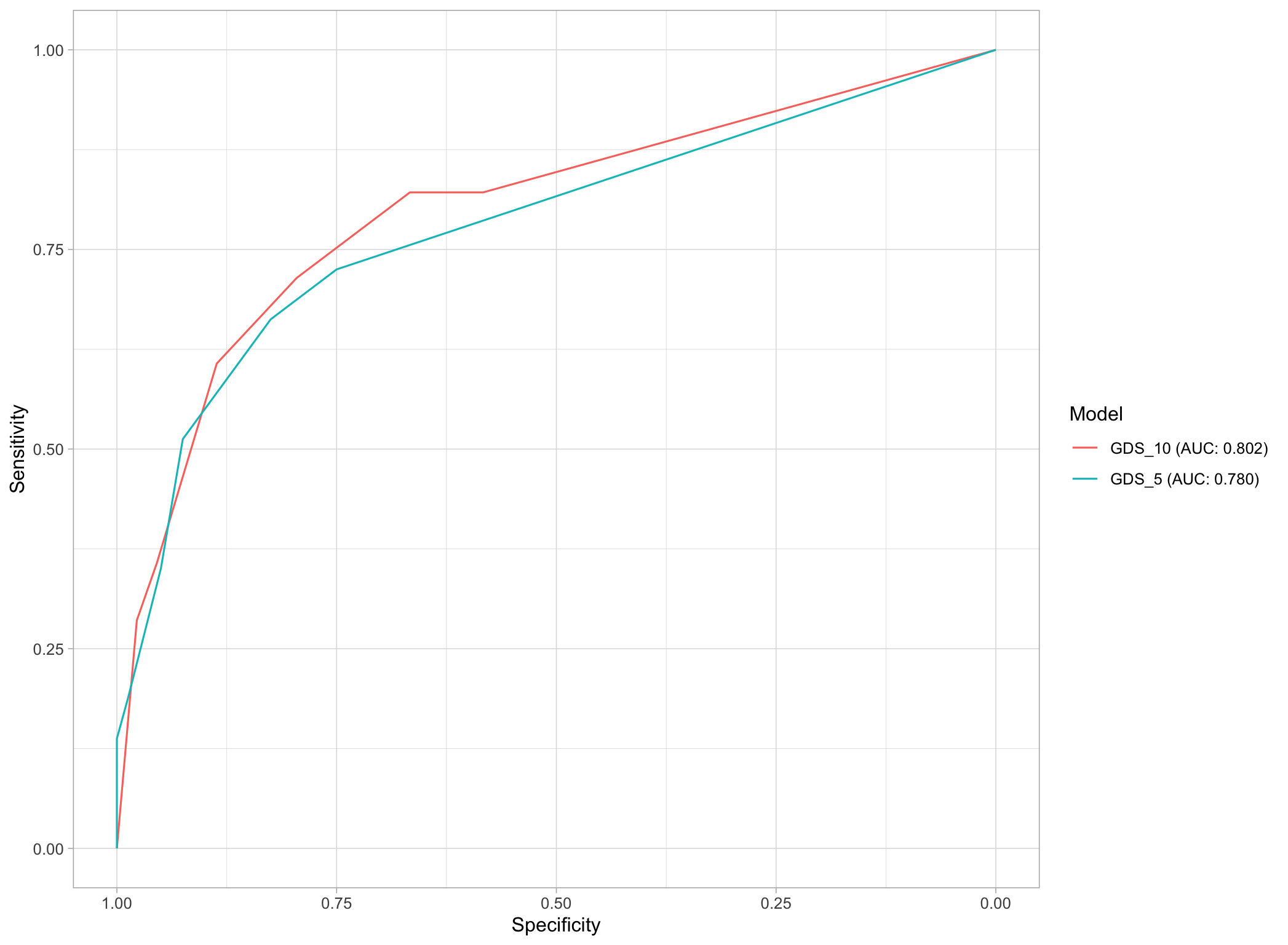

Supplement: Supplementary file 1 — Figure S1: Curves from GDS_5 validation model. Figure S2: Comparison of GDS_10 and GDS_5 ROC curves. [file AGM2-9--s001.docx]
